# Tract-specific MRI measures explain learning and recall differences in multiple sclerosis

**DOI:** 10.1093/braincomms/fcab065

**Published:** 2021-04-01

**Authors:** Mia Winter, Emma C Tallantyre, Thomas A W Brice, Neil P Robertson, Derek K Jones, Maxime Chamberland

**Affiliations:** 1 Department of Clinical Neuropsychology, University Hospital of Wales, Cardiff, CF14 4XW, UK; 2 Cardiff University Brain Research Imaging Centre, Cardiff University, Cardiff, CF24 4HQ, UK; 3 Division of Psychological Medicine and Clinical Neurosciences, Cardiff University School of Medicine, Cardiff, CF14 4XN, UK; 4 Helen Durham Centre for Neuroinflammation, University Hospital of Wales, Cardiff, CF14 4XW, UK; 5 Mary MacKillop Institute for Health Research, Australian Catholic University, Melbourne, Victoria 3000, Australia

**Keywords:** cognition, bundle load, Tractometry, lesionometry, structural reserve

## Abstract

Cognitive difficulties are common and a key concern for people with multiple sclerosis. Advancing knowledge of the role of white matter pathology in multiple sclerosis-related cognitive impairment is essential as both occur early in the disease with implications for early intervention. Consequently, this cross-sectional study asked whether quantifying the relationships between lesions and specific white matter structures could better explain co-existing cognitive differences than whole brain imaging measures. Forty participants with relapse-onset multiple sclerosis underwent cognitive testing and MRI at 3 Tesla. They were classified as cognitively impaired (*n* = 24) or unimpaired (*n* = 16) and differed across verbal fluency, learning and recall tasks corrected for intelligence and education (corrected *P*-values = 0.007–0.04). The relationships between lesions and white matter were characterized across six measures: conventional voxel-based T2 lesion load, whole brain tractogram load (lesioned volume/whole tractogram volume), whole bundle volume, bundle load (lesioned volume/whole bundle volume), Tractometry (diffusion-tensor and high angular resolution diffusion measures sampled from all bundle streamlines) and lesionometry (diffusion measures sampled from streamlines traversing lesions only). The tract-specific measures were extracted from corpus callosum segments (genu and isthmus), striato-prefrontal and -parietal pathways, and the superior longitudinal fasciculi (sections I, II and III). White matter measure-task associations demonstrating at least moderate evidence against the null hypothesis (Bayes Factor threshold < 0.2) were examined using independent *t*-tests and covariate analyses (significance level *P *<* *0.05). Tract-specific measures were significant predictors (all *P*-values < 0.05) of task-specific clinical scores and diminished the significant effect of group as a categorical predictor in Story Recall (isthmus bundle load), Figure Recall (right striato-parietal lesionometry) and Design Learning (left superior longitudinal fasciculus III volume). Lesion load explained the difference in List Learning, whereas Letter Fluency was not associated with any of the imaging measures. Overall, tract-specific measures outperformed the global lesion and tractogram load measures. Variation in regional lesion burden translated to group differences in tract-specific measures, which in turn, attenuated differences in individual cognitive tasks. The structural differences converged in temporo-parietal regions with particular influence on tasks requiring visuospatial-constructional processing. We highlight that measures quantifying the relationships between tract-specific structure and multiple sclerosis lesions uncovered associations with cognition masked by overall tract volumes and global lesion and tractogram loads. These tract-specific white matter quantifications show promise for elucidating the relationships between neuropathology and cognition in multiple sclerosis.

## Teaser

A key priority in multiple sclerosis is to uncover the neural mechanisms associated with cognitive change. Winter *et al*. demonstrate that the relationships between white matter pathology and the structure of specific tracts explain differences in learning and recall tasks in long-standing relapse-onset multiple sclerosis.

## Graphical Abstract

**Graphical Abstract:**
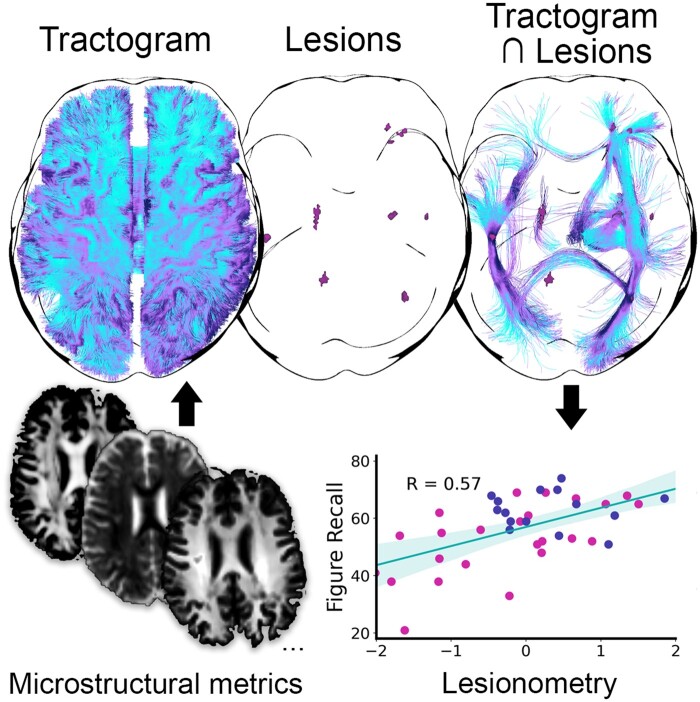

## Introduction

Cognitive impairment affects 50–75% of people with multiple sclerosis^1–3^ and is associated with adverse clinical outcomes and reduced quality of life.^4–6^ Whilst cognitive impairment in multiple sclerosis has been subject to increasing attention in the literature over recent decades, more work is needed to characterize cognitive phenotypes and particularly their relationships to neuropathology.^6–8^ Indeed, uncovering the precise neurobiological bases of cognitive deficits has been identified as a key research priority in multiple sclerosis.^8^

Cognitive impairment in multiple sclerosis is typically characterized by impaired information processing speed and memory, but a variety of tasks across many cognitive domains have demonstrated differences in multiple sclerosis cohorts compared to healthy controls.^1^^,^^2^^,^^6–10^ Even meta-analyses comprising large numbers of cases do not always agree on which types of tasks and/or cognitive domains demonstrate the most sensitivity in discriminating multiple sclerosis-related cognitive impairment.^9^^,^^11^^,^^12^ The variation in cognitive profiles^7^^,^^12^^,^^13^ perhaps mirrors the wide variation in the nature and location of neuropathology between individuals.^7^^,^^14^^,^^15^ However, the lack of agreed methodology for cognitive testing and classification of cognitive impairment are key challenges in unravelling the pathological correlates of cognitive impairment in multiple sclerosis.^8^

Another challenge is posed by the varied approaches to MRI, which is currently the best biomarker for both diagnosing and monitoring disease activity in multiple sclerosis.^16^ In keeping with the clinico-radiological paradox,^17^ traditional white matter measures such as T1 or T2 lesion loads correlate only modestly with cognition in multiple sclerosis^18^ and have not always performed well in predicting specific cognitive functions.^19^^,^^20^ While global measures such as lesion loads are valuable in clinical trials, these are not expected to explain inter-individual cognitive differences.^18^ Imaging approaches that uncover regional or structure-specific variation in pathology may perform better at providing evidence for clinical–pathological correlation.^21^ As the locus of pathology in the early stages of multiple sclerosis is predominantly in white matter^22–24^ with cognitive deficits also apparent early in the disease,^3^^,^^5^^,^^25^ white matter pathology may be a driver of cognitive deficits amenable to targeted rehabilitative or restorative interventions.^8^^,^^26–28^

Studies investigating the relevance of white matter pathology to cognition in multiple sclerosis have demonstrated the importance of lesion location,^29^ abnormalities in ‘diffusely abnormal’ and ‘normal-appearing’ white matter,^7^^,^^30^ and regional as well as tract-based microstructural abnormalities.^31–35^ Where diffusion-weighted imaging has been used, conventional diffusion-tensor measures such as fractional anisotropy (FA) predominate, with voxel-based analysis (e.g. tract-based spatial statistics) often employed.^33^ As demonstrated by recent approaches to tractography,^21^^,^^34–38^ more advanced approaches allow for enhanced anatomical and microstructural specificity.^39–44^ Combining state-of-the-art diffusion-MRI methods with examination of specific white matter structures and cognitive constructs (rather than composites from multiple cognitive domains) presents a novel opportunity for detailed characterization of the relationships between white matter structure and cognition in multiple sclerosis.^8^^,^^18^

We aimed to address these unmet needs by investigating whether individual differences in tract-specific measures can account for individual differences in performance on specific cognitive tasks between people with multiple sclerosis who are classified as cognitively impaired or unimpaired. We used novel methods^45^ to characterize individual tract macro- and microstructure and examined their performance, versus more global measures (e.g. conventional T2-weighted lesion load), in explaining cognitive group differences. We hypothesize that tract-specific volumetric and/or microstructural measures will perform better than the global imaging measures in explaining specific cognitive differences. Furthermore, we hypothesize that white matter measures differentially affected by lesion burden between groups may show some relative specificity in explaining performances in individual cognitive tasks.

## Materials and methods

### Estimate of sample size

The sample was derived from a larger clinical study into long-standing multiple sclerosis^46^ and *a priori* sample size was calculated from data (*n* = 26) involving a similarly defined cohort.^47^ Bester et al.^47^ examined tract-specific microstructural differences and compared those cognitively impaired (*n* = 10) and cognitively preserved (*n* = 16) using the same method for classifying cognitive impairment as applied in Tallantyre et al.^46^ They reported significant differences between groups in FA (*P *=* *0.02) and mean diffusivity (MD, *P *=* *0.01) within the splenium of the corpus callosum.^47^ Using the published means and standard deviations, the effect sizes were calculated, which were large (FA, *g*_Hedges_ = 1.2 and MD, *g*_Hedges_ = 1.3) with the impaired group demonstrating lower FA and higher MD.

These data were used to estimate sample size (with G*Power; www.gpower.hhu.de) for using a *t*-test to detect a white matter structural difference between cognitively impaired or unimpaired groups. Using the parameters of power 0.8, the smaller effect size 1.2, and alpha 0.05 for a two-tailed hypothesis, the minimum sample size derived for each group was *n* = 12. Similarly, the lower correlation (*r *=* *0.52, *P *=* *0.008) between corpus callosum (genu) FA and a cognitive task (verbal learning) reported by Bester et al. was used to estimate the sample size needed to examine structure-cognitive task correlations. Using the same power and alpha as above, the minimum whole sample size needed was *n* = 26.

### Participants

From 60 people with longstanding relapse-onset multiple sclerosis,^46^ 40 consented to undergo MRI within 12 months of their clinical assessment. Problematic clinical confounds (e.g. fatigue, depression, drug effects) were minimized by recruiting participants who demonstrated relatively low levels of disability and had never received disease modifying treatments.^48–51^ The study was approved by the Wales Research Ethics Committee 2 (16/WA/0051) and is in keeping with the principles of the Declaration of Helsinki.

### MRI acquisition

All scanning was performed using a Siemens PRISMA 3T system using a 32-channel receive-only radiofrequency head coil. All participants underwent the following sequences: (i) 3D T2 SPACE sequence^52^ where the flip angles of the train of refocusing pulses are optimized to increase the useable echo train duration (TR/TE: 3200/408 ms; voxel size: 1 × 1 × 1 mm^3^); (ii) 3D SPACE-FLAIR sequence (TR/TE: 5000/388 ms; TI: 1800 ms; voxel size: 1 × 1 × 1 mm^3^); (iii) 3D T1 MPRAGE (TR/TE: 2300/3 ms; flip angle: 9°; voxel size: 1 × 1 × 1 mm^3^); and (iv) high angular resolution diffusion imaging (HARDI; 14 *b* = 0 images; 30 directions at *b* = 1200 s/mm^2^; 60 directions at *b* = 2400 s/mm^2^; TR/TE: 9400/70 ms; voxel size: 2 × 2 × 2 mm^3^).

### Data pre-processing and derivation of white matter measures

Brain masks were extracted from the fluid attenuated inversion recovery (FLAIR) images using FSL *bet*.^53^ White matter lesion masks were semi-automatically delineated using 3D T2 and FLAIR images by a trained technician (blinded to the purpose of the study) using NeuROI (www.nottingham.ac.uk/research/groups/clinicalneurology/neuroi.aspx).

**Figure 1:**
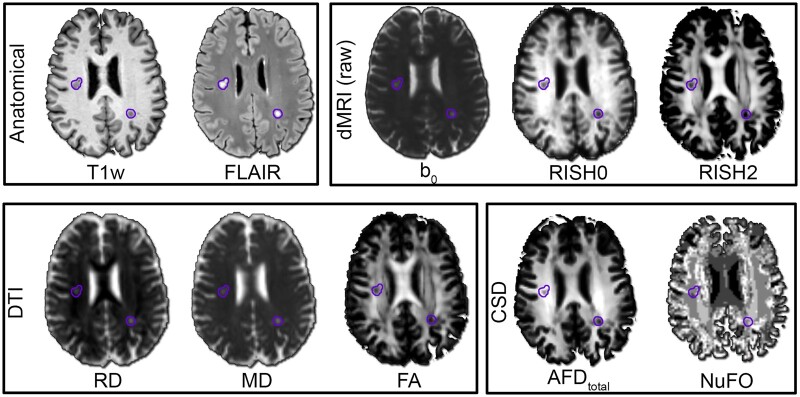
**Qualitative overview of the anatomical and diffusion maps derived for each subject.** RISH features (order 0 and 2) were derived using the highest *b*-value and represent the isotropic and anisotropic energy, respectively. Diffusion-tensor measures (RD, MD and FA) were derived using the lowest *b*-value. HARDI measures like the apparent fibre density (AFD) and the number of unique fibre orientations per voxel (NuFO) were derived from constrained spherical deconvolution. Hyperintense T2 lesions (FLAIR) are highlighted across the maps (purple outline).

Diffusion data were denoised^54^ and corrected for subject motion and distortion.^55^ To align anatomical images with diffusion data, the FLAIR images were warped to the upsampled *b* = 0 images from the diffusion MRI dataset using ANTs.^56^ The same transformation matrix was then applied to the brain mask and lesion mask. Diffusion tensors were generated using iteratively weighted least squares in MRtrix^57^ using only the *b* = 1200 s/mm^2^ data, followed by the derivation of FA, MD and radial diffusivity (RD) maps (Fig. 1). In addition, the total apparent fibre density (AFD) was derived from fibre orientation distribution functions^58^^,^^59^ obtained from multi-shell multi-tissue constrained spherical deconvolution^39^ using a single group response function. The number of fibre orientations (NuFO) in each voxel was also extracted.^60^ Finally, rotationally invariant spherical harmonics (RISH) features^61^ were derived for each subject using the *b* = 2400 s/mm^2^ shell (0th and 2nd orders only, RISH0 and RISH2, respectively). Briefly, RISH features capture the signal energy at a given shell and therefore, the higher the *b*-value, the more specific to intra-axonal space the RISH features are. Both orders capture different microstructural tissue properties; RISH0 captures the isotropic component of the signal, while RISH2 captures the variance in the signal, and therefore deviations from isotropy.

**Figure 2:**
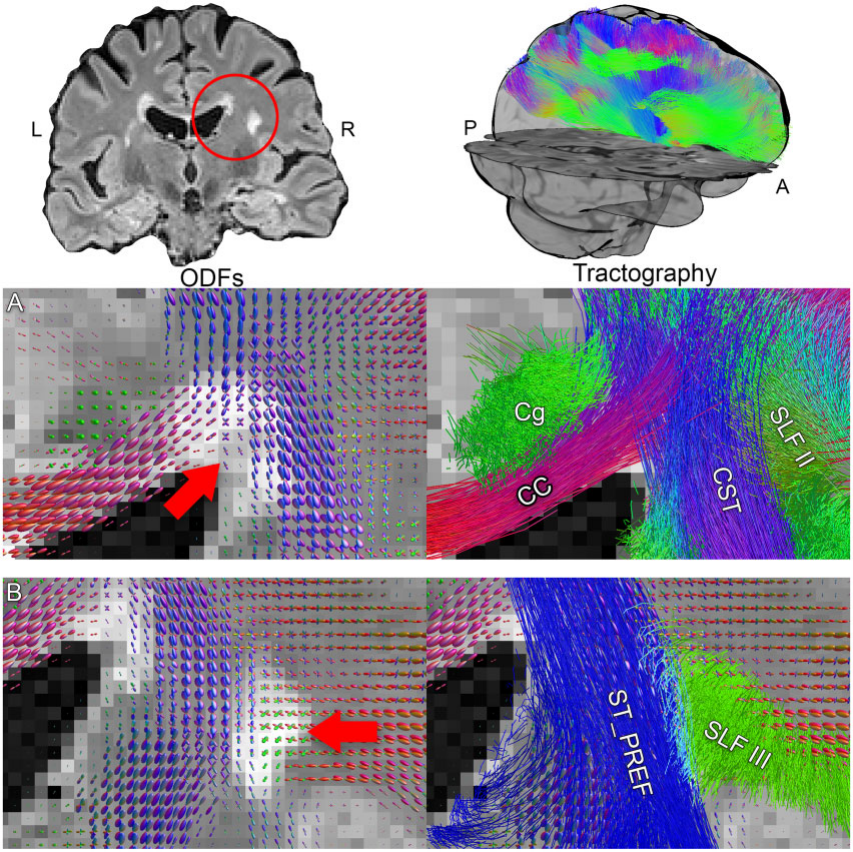
**Tractography results in a single participant.** Top row: Coronal view of the FLAIR image with lesions highlighted (red circle). On the right, 3D oblique view of the corona radiata and the superior longitudinal fasciculi in the vicinity of the lesion. (**A**) Fibre orientation distribution functions (fODFs) of the centrum semiovale (red arrow) shows continuity inside and around the lesion, suggesting preserved structural organization.^67^ On the right, multiple major pathways intersect and traverse the lesion (CST, corticospinal tract; Cg, Cingulum; CC, corpus callosum; SLF, superior longitudinal fasciculus). (**B**) A lesion adjacent to the striato-prefrontal connection (ST_PREF) shows preserved fibre orientations (red arrow) allowing reconstruction of the SLF-III pathway.

For each dataset, automated white matter tract segmentation was performed using TractSeg^42^ to obtain the following bilateral bundles of interest (Fig. 2): Corpus callosum (TractSeg sections 2 [genu] and 6 [isthmus]), striato-prefrontal pathway, striato-parietal pathway and superior longitudinal fasciculus (SLF, TractSeg sections I–III). These bundles were chosen in line with the white matter regions often associated with neuropathological burden in multiple sclerosis,^7^^,^^37^^,^^62^ the types of fibres affected by its pathology,^35^^,^^63^^,^^64^ and to achieve some coverage of anterior and posterior regions across commissural, projection, and association pathways. For each bundle, 2000 streamlines were generated. The microstructural measures were then averaged in each bundle. A whole brain tractogram was also derived for further analysis by concatenating all TractSeg outputs.

**Figure 3:**
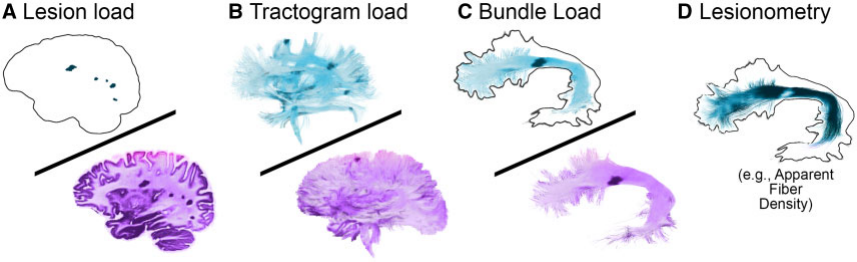
**Graphical overview of the white matter measures.** (**A**) conventional voxel-based lesion load (normalized by head size), (**B**) whole-brain tractogram load, whereby the volume of white matter intersecting with lesions is used as the numerator, (**C**) bundle load, which is the volume intersecting lesions divided by entire volume (example bundle: arcuate fasciculus) and (**D**) lesion-based Tractometry (lesionometry), where diffusion-MRI measures are sampled along the entire length of the streamlines that intersect lesions. Measures A–C range from 0 (0% interaction between lesions and white matter) to 1 (100% interaction between lesions and white matter). Visualization was performed using FiberNavigator.^76^

Next, white matter measures were derived (Fig. 3) according to Chamberland et al.^45^ These include (i) conventional T2 lesion load; (ii) whole brain tractogram load; and (iii) bundle load. In addition, (iv) a lesion-specific approach to the Tractometry^43^^,^^65^ framework was employed, where the diffusion MRI measures are sampled only within the portion of streamlines traversing a lesion. To reduce the high dimensionality of the data, two Tractometry factors were created from the tract-specific microstructural measures,^66^ one derived from whole bundle streamlines and the other from the streamlines affected by lesions only (henceforth referred to as the lesionometry factor; Fig. 3D).

### Behavioural measures

The sample was characterized using typical demographics, disease duration, years in education, Test of Premorbid Functioning UK^68^ (to estimate intelligence quotient [IQ]); Expanded Disability Status Scale^69^ (EDSS); Fatigue Assessment Instrument^70^ (FAI, 11-item severity scale); Multiple Sclerosis Impact Scale 29^71^ (MSIS29); and the Beck Depression Inventory II^72^ (BDI II).

The cognitive battery has been previously detailed^46^ and the same classification of cognitive impairment was applied (≥2 test scores ≤5th percentile against test normative data). However, only a subset of tasks from the original battery were considered for inclusion; those testing cognitive domains often impaired in multiple sclerosis (i.e. information processing speed, working memory, learning, recall, language and cognitive flexibility)^7^:

- The Paced Auditory Serial Addition Test^73^ (3″ version);
- Letter-Number Sequencing and Coding (Wechsler Adult Intelligence Scale IV)^74^;
- Story (immediate) Recall, Figure (immediate) Recall, List Learning (trials 1–5), Design Learning (trials 1–5), and Speed of Information Processing adjusted for motor speed (BIRT Memory and Information Processing Battery)^75^; and
- Verbal Fluency (Letter and Category) and Colour-Word Interference Test Conditions 3 and 4 (Delis-Kaplan Executive Function System).^77^

To examine if cognitive differences can be explained by variation in structural white matter properties, only those cognitive tasks demonstrating significant (*P *<* *0.05) differences between cognitively impaired and unimpaired groups following corrections for estimated IQ and years in education were included. The cognitive measures retained were Story Recall, Figure Recall, List Learning, Design Learning and Letter Fluency.

### Statistical analyses

All statistical analyses were completed using SPSS Statistics 26. The raw scores from the cognitive measures were used across analyses. Effect sizes are presented as *d* for *t*-tests, *r* for correlations, and *η_p_*2 for analyses of covariance and interpreted as large (LES), medium (MES) or small (SES).^78^

#### Tractometry factors

For the whole bundle Tractometry factors, the mean FA, MD, RD, AFD, RISH0, RISH2 and NuFO values underwent principal component analysis.^66^ The measures were standardized, the factors derived based on eigenvalues greater than 1, and any small coefficients were suppressed (absolute value below 0.3). The same method was used to create the lesionometry factors, but as derived from the within-bundle streamlines affected by lesions only (Fig. 3D). To ensure the data were suitable for principal component analysis, the Kaiser–Meyer–Olkin Measure of Sampling Adequacy (all ≥0.7) and Bartlett’s Test of Sphericity (all *P* < 0.0005) were applied.

#### Inclusion of cognitive measures

The cognitive measures considered for inclusion were compared between those cognitively impaired (*n* = 24) and those unimpaired (*n* = 16) using independent two-tailed *t*-tests. The tasks differing between groups (*P *<* *0.05) then underwent corrections for estimated IQ and years of education using analyses of covariance. The tasks still demonstrating a significant difference (*P *<* *0.05) between groups following these corrections were included.

#### Primary analyses

The white matter measures from the bilateral tracts included were reduced into single measures by averaging the left and right values. Consequently, there were seven bundles (corpus callosum sections 2 and 6, striato-prefrontal pathway, striato-parietal pathway, and SLF sections I, II and III) with four white matter measures associated with each tract (bundle volume, bundle load, whole bundle Tractometry factor and lesionometry factor). These white matter measures alongside lesion load and tractogram load were correlated against each task resulting in 30 correlations per task. Owing to the number of comparisons, whole sample (*n* = 40) Bayesian Pearson correlation was used to derive Bayes factors (BFs) to indicate the degree of evidence against the null hypothesis for each correlation (tolerance = 0.0001; maximum iterations = 2000; Monte Carlo samples = 10 000). At least moderate evidence (BF 0.1–0.33) for the alternative hypothesis was desired with the threshold for inclusion in subsequent analyses set to BF 0.2 so that any relationships would be closer to demonstrating strong (BF < 0.1), as opposed to anecdotal (BF > 0.33), evidence for the alternative hypothesis (note in SPSS evidence for null hypothesis are values >1 and evidence for alternative hypothesis are values <1).^79^ Only the surviving relationships per task were examined further to understand the nature of the relationships and to determine if these accounted for the task differences.

Analyses of covariance were used to gauge the impact of the retained white matter measures on the task-specific group differences. In each model, the cognitive task was the dependent variable, group the independent variable, and estimated IQ and years of education were covariates alongside the task-specific white matter measures. As the focus was on covariate effects, any heterogeneity of regression slopes or group-covariate interactions were of interest. The aim was to determine if the white matter measures (covariates) would diminish the effect of group as a significant categorical predictor of task scores. The significance level adopted for the influence of the covariates and for the effect of group was *P *<* *0.05. In addition to graphical linearity checks, the Levene’s Test of Equality of Error Variances was used to gauge any violations of the assumption of homogeneity of variances (*P *>* *0.05), and covariate collinearity checks (*r *<* *0.8) were applied when individual covariate effects were examined. Supplemental analyses conducted to elucidate the results included bivariate Pearson’s correlations and independent *t*-tests.

### Data availability

TractSeg is available at github.com/MIC-DKFZ/TractSeg. MRtrix is available at www.mrtrix.org/. FiberNavigator is available at github.com/chamberm/fibernavigator. The lesionometry toolbox will be available upon publication at github.com/chamberm/Lesionometry. The anonymized data and code used in the reported analyses are available on reasonable request from the first author.

## Results

### Sample

The 40 participants were 27 women and 13 men, mean age 58 years (range 44–78) and mean disease duration 27 years (range 15–47). Median Expanded Disability Status Scale at clinical assessment was 2.5 (range 0–6.0). Sample characteristics by group and whether these differed statistically are presented in Table 1.

**Table 1:** Sample characteristics by group with *P*-values (*<0.05) from independent *t*-tests except the Chi-Square for the sex ratios.

Characteristic Mean (SD)	Cognitively impaired *N* = 24	Not impaired *N* = 16	*P*-value
Age (years)	56.75 (8.45)	60.06 (7.39)	0.210
Sex (number of F:M)	15:9	12:4	0.630
Estimated IQ	105.86 (10.99)	113.37 (10.44)	0.037*
Years of education	14.71 (2.48)	16.06 (2.86)	0.120
Disease duration (years)	26.88 (7.09)	27.13 (9.37)	0.924
Disability status (EDSS)	2.85 (1.35)	3.00 (1.32)	0.737
Disease impact (MSIS29)	54.33 (25.11)	54.19 (21.98)	0.985
Depression (BDI II)	9.29 (10.41)	13.25 (12.98)	0.293
Fatigue severity (FAI)	3.40 (1.89)	3.74 (2.29)	0.604
Lesion load	0.55% (0.53%)	0.25% (0.23%)	0.018*
Tractogram load	47.2% (24.5%)	34.1% (22.9%)	0.096

*Note.* Estimated IQ and years of education were corrected for across analyses. IQ score (m = 100; SD = 15); EDSS <4 (low physical disability); MSIS29 (the higher the score, the greater the impact—score range 29–145); BDI II (scores 0–13 indicate minimal depression); Fatigue Severity <4 (unproblematic fatigue).

**Figure 4:**
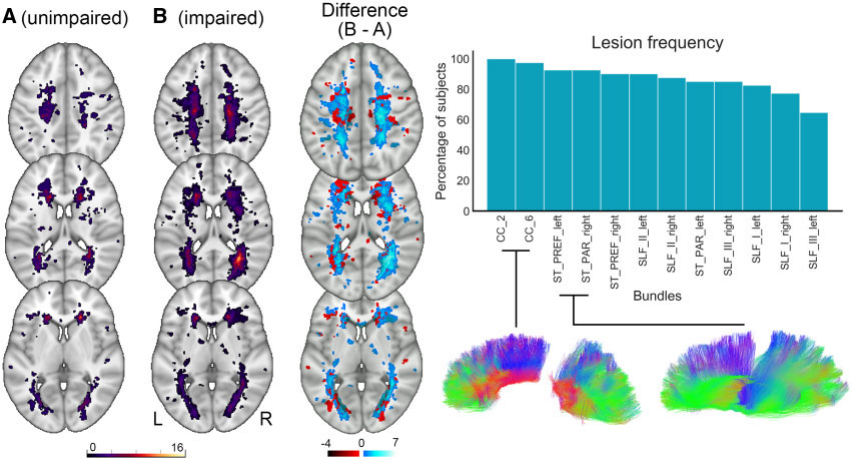
**Illustrations of lesion density and bundle-specific lesion frequencies.** Left: Density map of all white matter lesions across groups (cognitively unimpaired, cognitively impaired, and difference between groups). For both groups, the map shows voxels where a lesion was present in at least one of the participants. A higher number of lesions occurred within the temporoparietal region. Right: Lesion frequencies for the extracted bundles of interest, with the highest rates within the corpus callosum segments. CC, corpus callosum; ST_PREF, striato-prefrontal pathway; ST_PAR, striato-parietal pathway; SLF, superior longitudinal fasciculus.

Whole brain lesion load was significantly higher in the cognitively impaired group (Table 1) with right hemisphere lesion load particularly driving the difference [*t*(31.38) = 2.651, *P *=* *0.012, *d *=* *0.856, LES versus left hemisphere *t*(36.75) = 1.968, *P *=* *0.057, *d *=* *0.635, MES] between groups. The greatest lesion frequency difference appeared to localize to the right temporoparietal region (Fig. 4).

### Primary analyses

The datasets were complete across all tasks (*n* = 40) except Story Recall in which one datapoint was missing (*n* = 39).

#### Letter fluency

The Letter Fluency score difference between groups was moderate following corrections for estimated IQ and years in education [*F*(1,36) = 5.147, *P *=* *0.029, *η_p_*^2^ = 0.125]. However, as all white matter measure correlations demonstrated BFs in favour of the null hypothesis (BFs ranging from 2.4 to 8.1), no further analyses were conducted. These results suggest the white matter measures included did not account for the difference in Letter Fluency scores between groups.

#### Story recall

**Figure 5:**
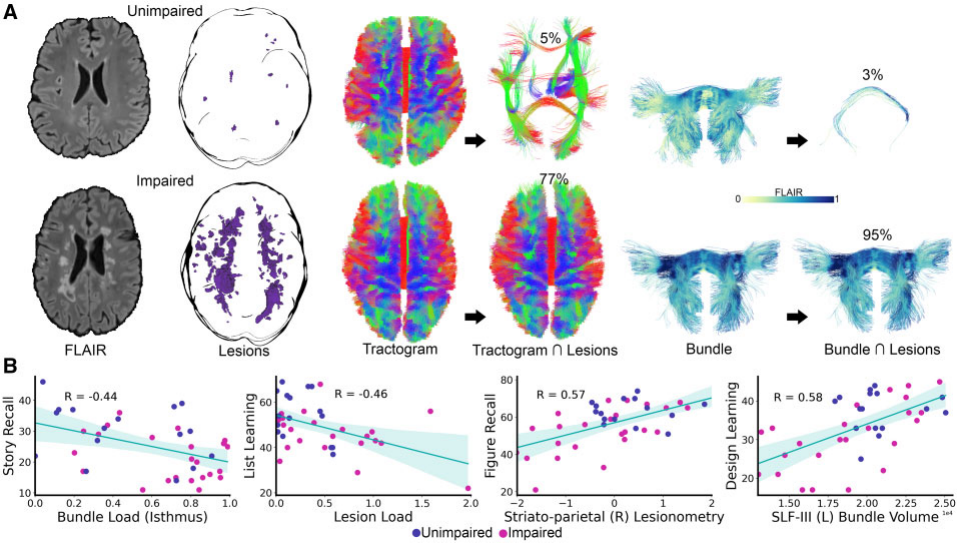
**Examples of lesion interactions with white matter measures and key task correlations.** (**A**) Examples of lesion interactions with tractogram load and bundle load. Top: Participant from the cognitively unimpaired group with 5% of their tractogram, and 3% of their isthmus bundle, affected by lesions. Below: Participant from the cognitively impaired group with 77% of their tractogram, and 95% of their isthmus, affected by lesions. The standard colour convention (i.e. red, green, and blue represent left-right, anterior-posterior, and inferior-superior orientations, respectively) is applied to the middle tractograms. Normalized FLAIR was used to colour-code the isthmus bundles on the right (0–1). (**B**) The key task-white matter measure correlations. Values from the cognitively impaired group in pink and from the unimpaired group, in purple. R, right; L, left.

The Story Recall score difference between groups was large following corrections for estimated IQ and years in education [*F*(1,35) = 6.359, *P *=* *0.016, *η_p_*^2^ = 0.154]. Of the white matter measures examined at whole sample level, only the isthmus bundle load correlation (*r* = −0.448, *BF *=* *0.138, *P *=* *0.004, MES) reached the BF threshold (see Fig. 5A for bundle and tractogram load illustrations and 5B for correlation scatterplot). When entered as a covariate in an analysis of covariance alongside estimated IQ and years of education, it was a unique predictor [*F*(1,34) = 5.628, *P *=* *0.023, *η_p_*^2^ = 0.142, LES] and rendered the large difference in scores between groups non-significant [*F*(1,34) = 2.589, *P *=* *0.117, *η_p_*^2^ = 0.071, MES]. The model as a whole accounted for 35.6% of variance in Story Recall scores [*F*(4,34) = 4.702, *P *=* *0.004, *η_p_*^2^ = 0.356, LES].

There was no group-isthmus bundle load interaction (*P *=* *0.977) with the correlations with Story Recall similar across groups (impaired: *r* = −0.344, *P *=* *0.108; unimpaired: *r* = −0.329, *P *=* *0.214). However, isthmus bundle load was significantly higher in the cognitively impaired group compared to the unimpaired group [impaired mean: 0.698, unimpaired mean: 0.454; *t*(38) = 2.649, *p *=* *0.012, *d *=* *0.855, LES].

#### List learning

The effect size for the List Learning score difference between groups following corrections for estimated IQ and years in education was moderate [*F*(1,36) = 5.367, *P *=* *0.026, *η_p_*^2^ = 0.130]. The only correlation to reach the BF threshold was with lesion load (*r* = −0.463, *BF *=* *0.091, *P* = 0.003, MES). When entered as a covariate alongside estimated IQ and years of education, it was a unique predictor [*F*(1,35) = 7.463, *P *=* *0.010, *η_p_*^2^ = 0.176, LES] and mitigated the score difference between groups [*F*(1,35) = 2.484, *P *=* *0.124, *η_p_*^2^ = 0.066, MES]. The model as a whole accounted for 29.5% of variance in List Learning scores [*F*(4,35) = 3.655, *P *=* *0.014, *η_p_*^2^ = 0.295, LES]. While the whole sample correlation was influenced by lesion load outliers from the cognitively impaired group (Fig. 5B), there were no outliers when lesion load values were examined by group.

There was no group-lesion load interaction (*P *=* *0.290) with the correlations with List Learning similar across groups (impaired: *r* = −0.420, *P *=* *0.041; unimpaired: *r* = −0.424, *P *=* *0.102). Lesion load was, however, higher in the cognitively impaired group than in the unimpaired (Table 1).

#### Figure recall

The Figure Recall score difference between groups was large following corrections for estimated IQ and years in education [*F*(1,36) = 8.084, *P *=* *0.007, *η_p_*^2^ = 0.183]. Multiple correlations reached the BF threshold (Table 2). Despite being significant predictors of variance in Figure Recall scores (white matter measure effects *P *<* *0.05), lesion load, tractogram load, the SLF II measures, and the striato-prefrontal measures did not attenuate the significant score difference between groups (all effects of group remained *P *<* *0.05). The bilaterally averaged SLF II and striato-prefrontal measures did not mask any significant unilateral differences between groups in these measures.

**Table 2:** Figure Recall-white matter measure Pearson correlations (*r*) surviving Bayes factor threshold <0.2 with group-measure differences (independent *t*-values) and interactions (*F*-values) presented.

Figure Recall						
Measure	Sample (*n* = 40) *r*	Bayes factor *BF*	Cognition impaired (*n* = 24) *r*	Cognition intact (*n* = 16) *r*	Group measure difference *t*	Group measure interaction *F*
Lesion Load	−0.509**	0.030	−0.452*	−0.272	2.481*	0.066
Tractogram L	−0.439**	0.152	−0.487*	−0.044	1.709	2.999
Isthmus BL	−0.456**	0.106	−0.522**	0.022	2.649*	5.546*
SLF I BL	−0.446**	0.131	−0.523**	−0.054	1.348	3.470
SLF I Tmtry	0.428**	0.188	0.489*	0.033	−1.052	2.024
SLF I Lmtry	0.477**	0.067	0.538**	−0.003	−1.296	2.609
SLF II Tmtry	0.443**	0.139	0.505*	−0.017	−1.439	3.000
SLF II Lmtry	0.461**	0.095	0.522**	−0.086	−1.690	3.574
St-Pref BL	−0.507**	0.032	−0.619**	−0.014	1.323	5.603*
St-Pref Tmtry	0.488**	0.051	0.640**	−0.181	−0.816	6.723*
St-Pref Lmtry	0.451**	0.119	0.640**	−0.468	−0.818	10.836**
St-Par BL	−0.472**	0.074	−0.497*	−0.094	2.052*	2.772
St-Par Tmtry	0.520**	0.023	0.561**	−0.042	−1.905	2.656
St-Par Lmtry	0.538***	0.014	0.556**	0.147	−2.055*	2.440

*Note.* The counterintuitive negative correlations of the striato-prefrontal Tractometry and lesionometry measures in the cognitively unimpaired, and the striato-prefrontal interactions, were owing to two cases (these were not outliers but when excluded the interactions disappeared). BL, bundle load; L, load; Lmtry, lesionometry; SLF, superior longitudinal fasciculus; St-Pref, striato-prefrontal pathway; St-Par, striato-parietal pathway; Tmtry, Tractometry.

* *P *<* *0.05,

** *P *<* *0.01,

*** *P *<* *0.0005.

In contrast, isthmus bundle load did mitigate the significant difference in Figure Recall scores between groups [*F*(1,35) = 3.963, *P *=* *0.054, *η_p_*^2^ = 0.102, MES], but did not explain unique variance in scores alongside estimated IQ and years in education (*P *=* *0.054). However, there was a group-isthmus bundle load interaction with both the bundle load and correlations differing between groups (Table 2). The model as a whole accounted for 31.2% of variance in Figure Recall scores [*F*(4,35) = 3.962, *P *=* *0.009, *η_p_*^2^ = 0.312, LES].

The SLF I measures were all significant predictors (*P *<* *0.05), but did not eradicate the group difference in scores until inputted together [effect of group *F*(1,33) = 3.987, *P *=* *0.054, *η_p_*^2^ = 0.108, MES]. These measures did not mask any significant unilateral differences between groups in these measures. Similarly, the bilaterally averaged striato-parietal measures (Table 2) were significant predictors (*P* < 0.05), but did not attenuate the effect of group until inputted together [*F*(1,33) = 3.988, *P *=* *0.054, *η_p_*^2^ = 0.108, MES]. However, both striato-parietal bundle load (impaired mean: 0.507; unimpaired mean: 0.324) and lesionometry (impaired mean: −0.244; unimpaired mean: 0.367) differed between groups (Table 2). These differences were underpinned by significant unilateral differences in right striato-parietal bundle load [impaired mean: 0.526, unimpaired mean: 0.318; *t*(38) = 2.276, *P* = 0.029, *d* = 0.735, MES] and lesionometry [impaired mean: −0.276, unimpaired mean: 0.415; *t*(38) = −2.253, *P* = 0.030, *d* = 0.727, MES]. While right striato-parietal bundle load (alongside estimated IQ and years in education) did not attenuate the significant effect of group (*p *=* *0.047), its lesionometry did [*F*(1,35) = 3.679, *P *=* *0.063, *η_p_*^2^ = 0.095, MES]. Right striato-parietal lesionometry was the only measure that accounted for both unique variance in Figure Recall scores [*F*(1,35) = 10.025, *P *=* *0.003, *η_p_*^2^ = 0.223, LES] and the significant group difference in scores (correlation with task *r *=* *0.573, *P *<* *0.0005, LES, *n* = 40; Fig. 5B). This model as a whole accounted for 40.4% of variance in Figure Recall scores [*F*(4,35) = 5.931, *P *=* *0.001, *η_p_*^2^ = 0.404, LES].

As tract-specific microstructural measures were meaningful here, these measures are provided from the whole right striato-parietal bundle and its lesionometry. With the former, the groups differed significantly on two measures, MD and RD (Table 3). Regarding the latter, the groups differed on four measures, MD, RD, AFD, and RISH0. All differences were in the anticipated directions i.e. MD and RD higher, and AFD and RISH0 lower, in the cognitively impaired group. The whole bundle factor loadings were as follows: RD (−0.987), RISH0 (0.982), MD (−0.960), AFD (0.955), RISH2 (0.937), NuFO (0.871) and FA (0.847). For the lesionometry factor, RD (−0.987), RISH0 (0.978), AFD (0.954) and MD (−0.950) similarly demonstrated the highest loadings followed by RISH2 (0.930), FA (0.831) and NuFO (0.773).

**Table 3:** Mean right striato-parietal microstructural differences between cognitively impaired and unimpaired groups (independent *t*-test).

Measure	Whole bundle		Lesioned streamlines	
	*t*	*P*	*t*	*P*
FA	−1.140	0.261	−1.976	0.055
MD	2.310	0.027*	2.083	0.044*
RD	2.278	0.029*	2.257	0.030*
AFD	−1.964	0.057	−2.472	0.018*
NuFO	−1.413	0.166	−1.503	0.141
RISH 0	−1.943	0.060	−2.061	0.046*
RISH 2	−1.457	0.153	−1.933	0.061

*Note.* MD and RD group means were higher, and FA, AFD, NuFO, RISH 0, and RISH 2 lower, in the cognitively impaired group than in those unimpaired. * significant at *P* <0.05.

#### Design Learning

The Design Learning score difference between groups was moderate following corrections for estimated IQ and years in education [*F*(1,36) = 4.549, *P *=* *0.040, *η_p_*^2^ = 0.112]. The correlations that reached the BF threshold were with genu bundle volume (*r *=* *0.486, *BF *=* *0.054, *P *=* *0.001, MES), SLF III bundle volume (*r *=* *0.602, *BF *=* *0.002, *P *<* *0.0005, LES), and striato-prefrontal bundle volume (*r *=* *0.477, *BF *=* *0.067, *P *=* *0.002, MES). When entered alongside estimated IQ and years in education as covariates, neither genu nor striato-prefrontal bundle volumes (despite being significant predictors *P *<* *0.05) accounted for the significant difference in scores between groups (effect of group *P *=* *0.024 and *P *=* *0.015, respectively). The groups did not differ in these white matter measures, but the whole sample correlations again appeared driven by the cognitively impaired group (genu impaired: *r *=* *0.623, *P *=* *0.001, unimpaired: *r *=* *0.002, *P *=* *0.994; striato-prefrontal impaired: *r *=* *0.645, *P *=* *0.001, unimpaired: *r*=-0.134, *P *=* *0.621).

When the SLF III bundle volume was entered as a covariate alongside estimated IQ and years of education, it was a unique predictor [*F*(1,35) = 10.074, *P *=* *0.003, *η_p_*^2^ = 0.223, LES] and rendered the score difference between groups non-significant [*F*(1,35) = 3.227, *P *=* *0.081, *η_p_*^2^ = 0.084, MES]. The model as a whole accounted for 46.9% of variance in Design Learning scores [*F*(4,35) = 7.742, *P *<* *0.0005, *η_p_*^2^ = 0.469, LES]. There was no group-SLF III bundle volume interaction (*P *=* *0.154), but this bundle volume did differ between groups [impaired mean: 22001.1, unimpaired mean: 24047.5; *t*(37.8) = −2.210, *P *=* *0.033, *d *=* *0.713, MES], which was underpinned by a significant difference in left hemisphere SLF III bundle volume [impaired mean: 18797.6, unimpaired mean: 20736.4; *t*(37.4) = −2.156, *P *=* *0.038, *d *=* *0.696, MES]. Indeed, this left-sided bundle volume alone (with estimated IQ and years in education also as covariates) both uniquely predicted [*F*(1,35) = 9.029, *P *=* *0.005, *η_p_*^2^ = 0.205, LES] Design Learning scores and eradicated the significant score difference [*F*(1,35) = 3.187, *P *=* *0.083, *η_p_*^2^ = 0.083, MES] between groups (correlation with task *r *=* *0.580, *P *<* *0.0005, LES, *n* = 40; Fig. 5B). This model as a whole accounted for 45.7% of variance in Design Learning scores [*F*(4,35) = 7.360, *P *<* *0.0005, *η_p_*^2^ = 0.457, LES]. Both whole sample task-SLF III bundle volume correlations were seemingly influenced by the cognitively impaired group (bilaterally averaged, impaired: *r *=* *0.643, *P *=* *0.001, unimpaired: *r *=* *0.154, *P *=* *0.569; left-sided, impaired: *r *=* *0.631, *P *=* *0.001, unimpaired: *r *=* *0.060, *P *=* *0.826).

## Discussion

The mapping of region-specific and tract-specific structural differences with cognitive differences in multiple sclerosis remains underutilized for recognizing individual differences, predicting functional outcomes, optimizing disease monitoring, and supporting treatment options. Here we have demonstrated that, in a cohort with long-standing multiple sclerosis, relatively low physical disability and functional impact (Table 1), four out of five cognitive differences were explained by variation in white matter measures. Furthermore, three cognitive differences were better explained by tract-specific than global measures. We have demonstrated that variation in pathological burden reflected by both macro- and microstructural tract-specific measures underpinned these cognitive group differences.

Despite the groups being well matched across demographic and clinical variables, cognitive differences were demonstrated along with greater lesion burden in those classed as cognitively impaired (Table 1). Indeed, whilst tractogram load did not differ between groups, on average 47% of the tractogram was affected by lesions in the cognitively impaired group in contrast to the 34% in those unimpaired. Overall, these global measures were less meaningful as predictors of cognitive differences than tract-specific measures, but tractogram load nonetheless supplements lesion load by allowing for convenient quantification and illustration of the structural network interacting with lesions, beyond that afforded by conventional 2D slice-based visualizations (Fig. 5A).

### Verbal tasks

None of the white matter imaging measures selected for this study accounted for the difference in Letter Fluency between groups. The group difference in List Learning, in turn, was the only one explained by a global imaging measure (lesion load), which is in keeping with previous studies linking verbal learning with lesion load in relapsing-remitting multiple sclerosis.^80^^,^^81^ In contrast, both verbal and visual immediate recall score differences were accounted for by the isthmus of the corpus callosum. It appears the pathological burden within the isthmus (mean bundle load 60%, *n* = 40) was the key driver of the adverse impact on these task scores as opposed to the isthmus being the most critical structure supporting these functions. While temporoparietal regions are involved in recall^82^ and the isthmus likely contributes, visual recall was better predicted by the striato-parietal pathways and verbal recall has previously been associated with other white matter tracts^83–85^ not considered in this study.

### Visual tasks

Right striato-parietal lesionometry and isthmus bundle load were the only measures that, by themselves, mitigated the group difference in Figure Recall. The mean bundle loads of both these tracts differed between groups (isthmus: impaired 70%, unimpaired 45%; right striato-parietal: impaired 53%, unimpaired 32%) suggesting a critical role for difference in tract-specific pathological burden in explaining the group difference in Figure Recall. In addition to bundle load, Tractometry and leisonometry featured particularly in association with Figure Recall. In fact, the lesionometry correlations were the highest among the SLF I, SLF II, and striato-parietal measures surviving the BF threshold (Table 2). Therefore, the interaction of lesions with the microstructure of the streamlines traversing them was especially relevant to Figure Recall performance. Having reduced multiple microstructural measures using principal component analysis, we uncovered neuropathological effects on measures such as AFD and RISH0 (alongside RD and MD) in lesionometry, which have not been as studied as e.g. FA in multiple sclerosis, but which differed between groups and were among the highest loading measures across factors meaningful for visual recall (Table 3).

Visual learning, in turn, was particularly associated with striato-prefrontal, genu, and SLF III whole bundle volumes, with only the latter, and the left SLF III volume specifically, eradicating the significant score difference between groups. There were no group differences in SLF III bundle loads and so the group differences in the bilaterally averaged, and left, SLF III volumes may reflect neuropathology extending beyond the streamlines directly interacting with lesions. Together these results suggest that the pathological burden on tracts converging in parietal regions was particularly meaningful in accounting for group differences in visuospatial-constructional tasks i.e. Figure Recall and Design Learning. This, in turn, is congruent with previous associations between posterior temporal and/or parietal regions and performance in these types of task.^86^^,^^87^

### Key contributions

Periventricular and posterior regions have often been associated with neuropathological burden in multiple sclerosis with some having demonstrated structural differences between multiple sclerosis groups in these regions previously, particularly within the posterior corpus callosum.^7^^,^^47^^,^^64^ However, we have enhanced these previous findings by linking variation in several structures to differences in specific cognitive functions. Our results have demonstrated that both (task-relevant) regional white matter and tract-specific structural reserve are important in understanding cognitive differences in multiple sclerosis, even in samples considered to have low disease impact (Table 1). We highlight that quantifying structural reserve benefits from specificity not provided by global measures such as lesion load or tractogram load, or even whole bundle measures, which can mask the relationships between individual tracts and specific cognitive differences. Furthermore, considering the differences in white matter measure correlations between groups across the visual tasks, the potential cumulative effects of tract-specific neuropathological variation across task-relevant structures may underpin these group differences by relatively better structural reserve facilitating better functional (i.e. cognitive) reserve. This, in turn, may mask relevant structure-function relationships in those cognitively unimpaired.^88–90^ Whilst it is important to acknowledge the relatively small group sizes, this pattern of differing correlations was repeated across all measure-visual task relationships with the cognitively impaired group driving the strengths of the whole sample correlations. It is worth noting that despite only a few white matter measures demonstrating statistically significant group differences, across the tracts included in this study, all measure group means favoured the cognitively unimpaired group with only two exceptions (SLF II bundle volume and genu lesionometry).

### Limitations

Whilst we have demonstrated that cognitive group differences can be explained by neuropathology in white matter, we are not suggesting that cortical influences can be discounted. The potential concomitant effects derived from cortical structures are unknown, and future work including cortical variables alongside these measures may elucidate their relationships and respective contributions to behaviour. Another consideration is that among the white matter measure-cognitive task relationships that did not reach the BF threshold, there may have been some meaningful unilateral correlations that were masked by the averaging of the right and left values from the striato-cortical and SLF pathways. Owing to asymmetries in lesion distributions across bilateral tracts, future work could uncover further unilateral tract-specific influences on particular cognitive tasks. A further limitation is the approach taken to classify cognitive impairment as this allowed for there to be ‘normal’ and ‘impaired’ performances in the impaired and unimpaired groups, respectively, at the level of the individual task. However, this was somewhat mitigated by the focus being on tasks that differed between groups. Last, as the bundles were selected owing to traversing regions often associated with neuropathology in multiple sclerosis, rather than according to the neuroanatomy associated with specific cognitive tasks, it is not known if other white matter structures may have contributed to performance differences.

## Conclusion

These results highlight the potential for diffusion-weighted MRI to disentangle the relationships between regional neuropathology and performance on specific cognitive tasks in multiple sclerosis. The benefits for clinical practice include the ability of this approach to better measure eligibility and the effects of targeted therapeutic and rehabilitative interventions, allowing also for greater precision in measurement of outcomes. It may be that the measures examined in this study can offer superior pathological specificity to clinically relevant processes, in which case new treatment effects can be uncovered and quantified. However, it is not yet clear when one measure may demonstrate better sensitivity than another and future work could help inform how to optimize their application.

## Abbreviations

AFD =: apparent fibre density
BF =: Bayes factor
FA =: fractional anisotropy
FLAIR =: fluid attenuated inversion recovery
IQ =: intelligence quotient
LES =: large effect size
MD =: mean diffusivity
MES =: moderate effect size
MPRAGE =: magnetization prepared rapid gradient echo
MSIS29 =: Multiple Sclerosis Impact Scale 29
NuFO =: number of fibre orientations
RD =: radial diffusivity
RISH =: rotationally invariant spherical harmonics
SES =: small effect size
SLF =: superior longitudinal fasciculus
TE/TI/TR =: echo time/inversion time/repetition time
